# Enhanced Recovery Care vs. Traditional Care in Laparoscopic Hepatectomy: A Systematic Review and Meta-Analysis

**DOI:** 10.3389/fsurg.2022.850844

**Published:** 2022-03-22

**Authors:** Xueyin Zhou, Xueyi Zhou, Jiasheng Cao, Jiahao Hu, Win Topatana, Shijie Li, Sarun Juengpanich, Ziyi Lu, Bin Zhang, Xu Feng, Jiliang Shen, Mingyu Chen

**Affiliations:** ^1^School of Medicine, Wenzhou Medical University, Wenzhou, China; ^2^Department of Nursing, Sir Run-Run Shaw Hospital, Zhejiang University, Hangzhou, China; ^3^Department of General Surgery, Sir Run-Run Shaw Hospital, Zhejiang University, Hangzhou, China; ^4^Zhejiang University School of Medicine, Zhejiang University, Hangzhou, China; ^5^College of Pharmaceutical Sciences, Zhejiang University, Hangzhou, China

**Keywords:** enhanced recovery after surgery (ERAS), traditional care, laparoscopic hepatectomy (LH), meta-analysis, systematic review

## Abstract

**Background:**

Enhanced recovery care could alleviate surgical stress and accelerate the recovery rates of patients. Previous studies showed the benefits of enhanced recovery after surgery program in liver surgery, but the exact role in laparoscopic hepatectomy is still unclear.

**Aim:**

We aimed to perform a meta-analysis to evaluate the safety and efficacy of enhanced recovery after a surgery program in laparoscopic hepatectomy.

**Methods:**

The relative studies from a specific search of PUBMED, EMBASE, OVID, and Cochrane database from June 2008 to February 2022 were selected and included in this meta-analysis. The primary outcomes included length of hospital stay, duration to functional recovery, and overall postoperative complication rate. The secondary outcomes included operative time, intraoperative blood loss, cost of hospitalization, readmission rate, Grade I complication rate, and Grade II–V complication rate.

**Results:**

A total of six studies with 643 patients [enhanced recovery care (*n* = 274) vs. traditional care (*n* = 369)] were eligible for analysis. These comprised three randomized controlled trials and three retrospective studies. Enhanced recovery care group was associated with decreased hospital stay [standard mean difference (SMD) = −0.56, 95% confidence interval (CI) = −0.83~−0.28, *p* < 0.0001], shorter duration to functional recovery (SMD = −1.14, 95% CI = −1.92~−0.37, *p* = 0.004), and lower cost of hospitalization Mean Difference (MD) = −1,539.62, 95% CI = −1992.85~−1086.39, *p* < 0.00001). Moreover, a lower overall postoperative complication rate was observed in enhanced recovery care group [Risk ratio (RR) = 0.64, 95% CI = 0.51~0.80, *p* < 0.0001] as well as lower Grade II–V complication rate (RR = 0.55, 95% CI = 0.38~0.80, *p* = 0.002), while there was no significant difference in intraoperative blood loss (MD = −65.75, 95% CI = −158.47~26.97, *p* = 0.16), operative time (MD = −5.44, 95% CI = −43.46~32.58, *p* = 0.78), intraoperative blood transfusion rate [Odds ratio (OR) = 0.71, 95% CI = 0.41~1.22, *p* = 0.22], and Grade I complication rate (RR = 0.73, 95% CI = 0.53~1.03, *p* = 0.07).

**Conclusion:**

Enhanced recovery care in laparoscopic hepatectomy should be recommended, because it is not only safe and effective, but also can accelerate the postoperative recovery and lighten the financial burden of patients.

## Introduction

Enhanced recovery after surgery (ERAS) was first introduced by Kehl et al. (1, 2) in colorectal surgery during the 1990's. After the implementation of ERAS in colorectal surgery, it was soon recommended for other types of surgeries and revolutionized the conventional perioperative patterns. ERAS is a multimodal, evidence-based approach aiming to optimize patient care during perioperative care (3). ERAS can attenuate the physical and psychological stress responses and complications during peri-operation *via* a series of optimization measures, such as preoperative education, perioperative fluid management, minimally invasive techniques, optimal pain control, and early initiation of oral feeding (4–7). Over the past 10 years, ERAS has been rapidly applied in surgery, including gastric (8–10), urologic (11, 12), vascular (13, 14), gynecologic (15), and hepatic procedures (16–19).

Recent years have witnessed a brisk development in laparoscopic hepatectomy involving less stress and trauma compared to open surgery. It has merits of less morbidity associated with a lengthy incision, shorter length of hospital stay (LOS), earlier recovery of function, and less post-operative pain (20, 21). Considering that the recommendation of ERAS was rarely reported in laparoscopic hepatectomy, it is suspicious that the ERAS program is suitable for patients undergoing laparoscopic hepatectomy. Moreover, previous studies reported that patients receiving ERAS were associated with the accelerated recovery and shorter LOS than those receiving traditional care (TC) in open hepatectomy (17, 22). As laparoscopic hepatectomy is widely applied in clinical practice, it is necessary to explore the exact role of ERAS in laparoscopic hepatectomy.

In the study, we performed a meta-analysis to get a comprehensive understanding of the efficacy and safety of ERAS in laparoscopic hepatectomy compared to TC.

## Materials and Methods

This meta-analysis has adhered to the guidelines of Preferred Reporting Items for Systematic Reviews and Meta-Analyses (PRISMA) (23).

### Study Selection

Two of the authors (Dr. Chen and Dr. Zhou) performed the meta-analysis search independently, using PUBMED, EMBASE, OVID, and Cochrane database. The search was performed to identify all studies comparing ERAS and Non-ERAS from June 2008 to February 2022. The search strategy was based on the following index words: “enhanced recovery after surgery,” “enhanced recovery,” “ERAS,” “fast track,” “fast-track,” “accelerated recovery,” “laparoscopic liver resections,” “laparoscopic liver resection,” “laparoscopic hepatectomy,” and “hand-assisted laparoscopic hepatectomy.” Only studies on humans and in English were considered for inclusion. Reference lists of all retrieved articles were manually searched for additional studies.

### Selection Criteria and Exclusion Criteria

The inclusion criteria were as follows: (1) Comparison of the primary outcome of ERAS and non-ERAS (including LOS, duration to functional recovery, and overall postoperative complication rate) in laparoscopic hepatectomy; (2) reporting the secondary outcome of ERAS and non-ERAS (including operative time, intraoperative blood loss, cost of hospitalization, readmission rate, grade I complication rate, and grade II–V complication rate) in laparoscopic hepatectomy; and (3) if dual studies were reported by the same institution or authors, only the most recent publication or the highest quality of study was included.

The exclusion criteria were as follows: (1) The outcomes of ERAS and TC were not compared; (2) patients did not undergo laparoscopic hepatectomy; (3) studies without full text; and (4) those without clear outcomes.

### Data Extraction and Assessment of Risk of Bias

Two of the authors (Dr. Chen and Dr. Zhou) independently performed data extraction. If any disagreement existed, the third author (Dr. Cao) was involved in data extraction and discussion until a consensus was reached. The parameters for each study were as follows: (1) First author, publication year; (2) the number and characteristics of patients; (3) the primary outcomes, including LOS, overall postoperative complication rate, and duration to functional recovery; and (4) the secondary outcomes, including operative time, intraoperative blood loss, intraoperative blood transfusion rate, Grade I complication rate, Grade II–V complication rate, the cost of hospitalization, and readmission rate.

Two of the authors (Dr. Chen and Dr. Zhou) independently assessed the risk of bias. We used Risk of bias tool (RoB2) and ROBINS-I (Risk of Bias in Non-randomized Studies of Interventions) to assess the quality of randomized clinical trials (RCTs) and non-RCTs, respectively. Additionally, GRADEpro Guideline Development Tool (GDT) was also used to evaluate every outcome in our meta-analysis. If any disagreement existed, the third author (Dr. Cao) was involved in data extraction and discussion until a consensus was reached (Supplementary Material).

### Statistical Analysis

This meta-analysis was performed by Review Manager (RevMan, Version 5.4). Continuous outcomes were analyzed using the estimation of weighted mean difference (WMD) or standard mean difference (SMD). Dichotomous outcomes were analyzed using the estimation of odds ratio (OR) or risk ratio (RR). Results were presented with 95% confidence intervals (CIs). If the original text manifest median (interquartile range) or median (range), we calculated the mean ± SD *via* the algorithm provided by Luo et al. (23) and Wan et al. (24). A random-effect model was used if heterogeneity was considered statistically significant (*p* < 0.05 or *I* > 50%). Otherwise, there was no heterogeneity and a fixed-effect model was used. A value of *p* < 0.05 was deemed as statistically significant.

## Results

### Selection of Studies

**Figure 1:**
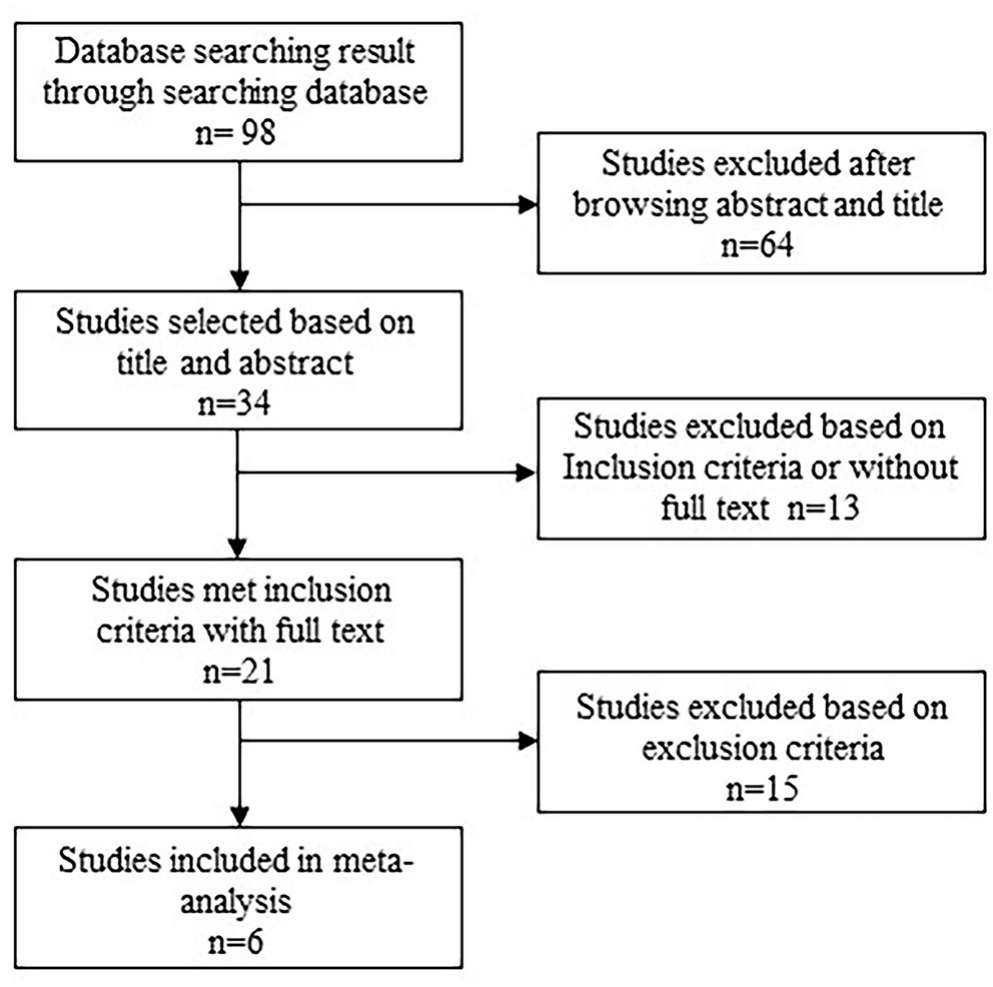
Flowchart showing selection of studies included in the meta-analysis.

A total of 20 studies initially met the inclusion criteria, in which 10 studies did not involve laparoscopic hepatectomy, 3 studies were reported by the same institution or author, and 1 study did not compare ERAS and TC. Finally, a total of six studies published between 2009 and 2018 were included in the study, which was conducted on 643 patients in the ERAS group (*n* = 274) and TC group (*n* = 369). The flow chart of retrieval is shown in Figure 1. The characteristics of patients in the six studies are shown in Table 1.

**Table 1:** Baseline characteristics of studies included in the meta-analysis.

**First author**	**Study type**	**Year**	**Number**		**Age**		**Sex, M/F**		**ASA I/II/III/IV**		**Malignant/Benign**		**Mortality**	
			**ERAS**	**TC**	**ERAS**	**TC**	**ERAS**	**TC**	**ERAS**	**TC**	**ERAS**	**TC**	**ERAS**	**TC**
Jan H. Stoot	CCT	2009	13	13	55 (34–82)	45 (26–70)	3/10	2/11	3/9/1/0	6/6/1/0	5/8	2/11	0	0
Belinda Sánchez-Pérez	CCT	2012	26	17	58.3 (29–77)	52.5 (29–84)	15/11	10/7	0/13/13/0	0/9/8/0	12/14	3/14	0	0
F He	RCT	2015	48	38	56.3 ± 16.3	60.4 ± 20.7	22/26	18/20	10/26/2/0	12/24/2/0	31/17	24/14	0	0
Xiao Liang	RCT	2016	80	107	53.4 ± 13.5	55.5 ± 12.8	37/43	50/57	35/45/0/0	49/58/0/0	51/29	55/52	0	0
Yuan Ding	CCT	2018	49	133	56.04 ± 11.50	56.31 ± 11.57	31/18	88/45	NR	NR	33/16	99/34	0	0
Xiao Liang	RCT	2018	58	61	58 (16–80)	59 (37–85)	25/33	22/39	12/35/11	8/48/5/0	29/29	44/17	0	0

*ASA, American Society of Anesthesiologists; CCT, case control study; ERAS, enhanced recovery after surgery; F, female; M, male; NR, not reported; RCT, randomized clinical trial; TC, traditional care*.

### Primary Outcomes

#### Length of Hospital Stay

**Figure 2:**
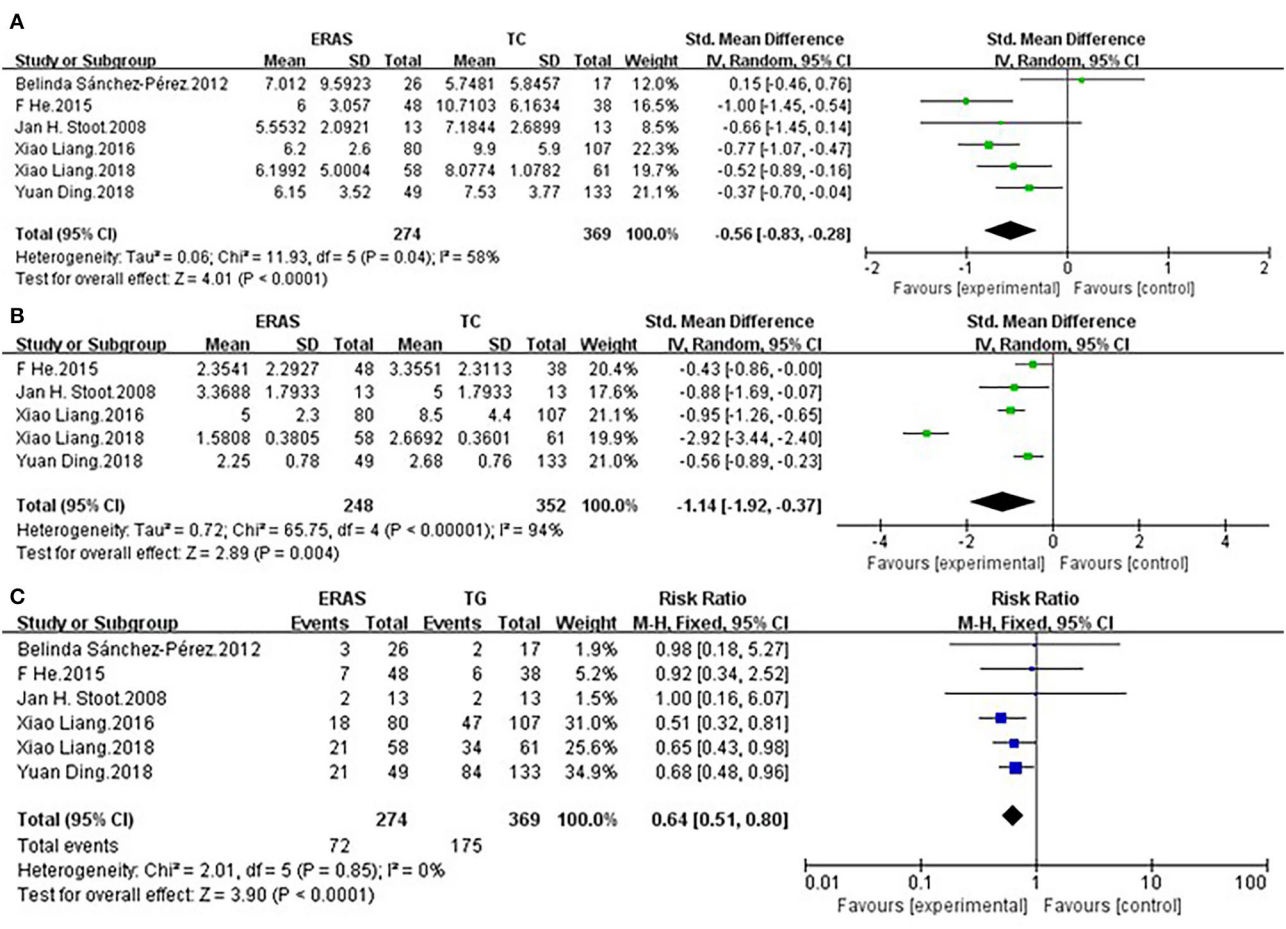
Comparisons of primary outcomes after enhanced recovery after surgery (ERAS) or traditional care (TC) in patients undergoing laparoscopic hepatectomy. The differences in **(A)** length of hospital stay (LOS), **(B)** duration to functional recovery, and **(C)** overall postoperative complication rate.

All studies (25–30) reported the LOS. LOS of ERAS group (*n* = 274) was significantly shorter than that of TC group (n = 369) (SMD = −0.56, 95% CI = −0.83~−0.28, *p* < 0.0001). There was no significant heterogeneity among the six studies, and a random-effect model was used (I^2^ = 58%, *p* = 0.04) (Figure 2A).

#### Duration to Functional Recovery

Five studies (25–28, 30) containing 600 patients reported duration to functional recovery. The ERAS group (n = 248) showed significant reduction of the time to functional recovery when compared to the TC group (*n* = 352) (SMD = −1.14, 95% CI: −1.92~−0.37, *p* = 0.004). A random-effect model was used on account of significant heterogeneity (*I*^2^ = 94%, *p* < 0.00001) (Figure 2B).

#### Overall Postoperative Complication Rate

All studies (25–30) reported overall postoperative complication rate. The ERAS group (n = 274) showed significant reduction of the overall postoperative complication rate when compared to the TC group (*n* = 369) (RR = 0.64, 95% CI = 0.51~0.80, *p* < 0.0001). There was no heterogeneity among the six studies, and a fixed-effect model was used (*I*^2^ = 0%, *p* = 0.85) (Figure 2C).

### Secondary Outcomes

#### Operative Time

**Figure 3:**
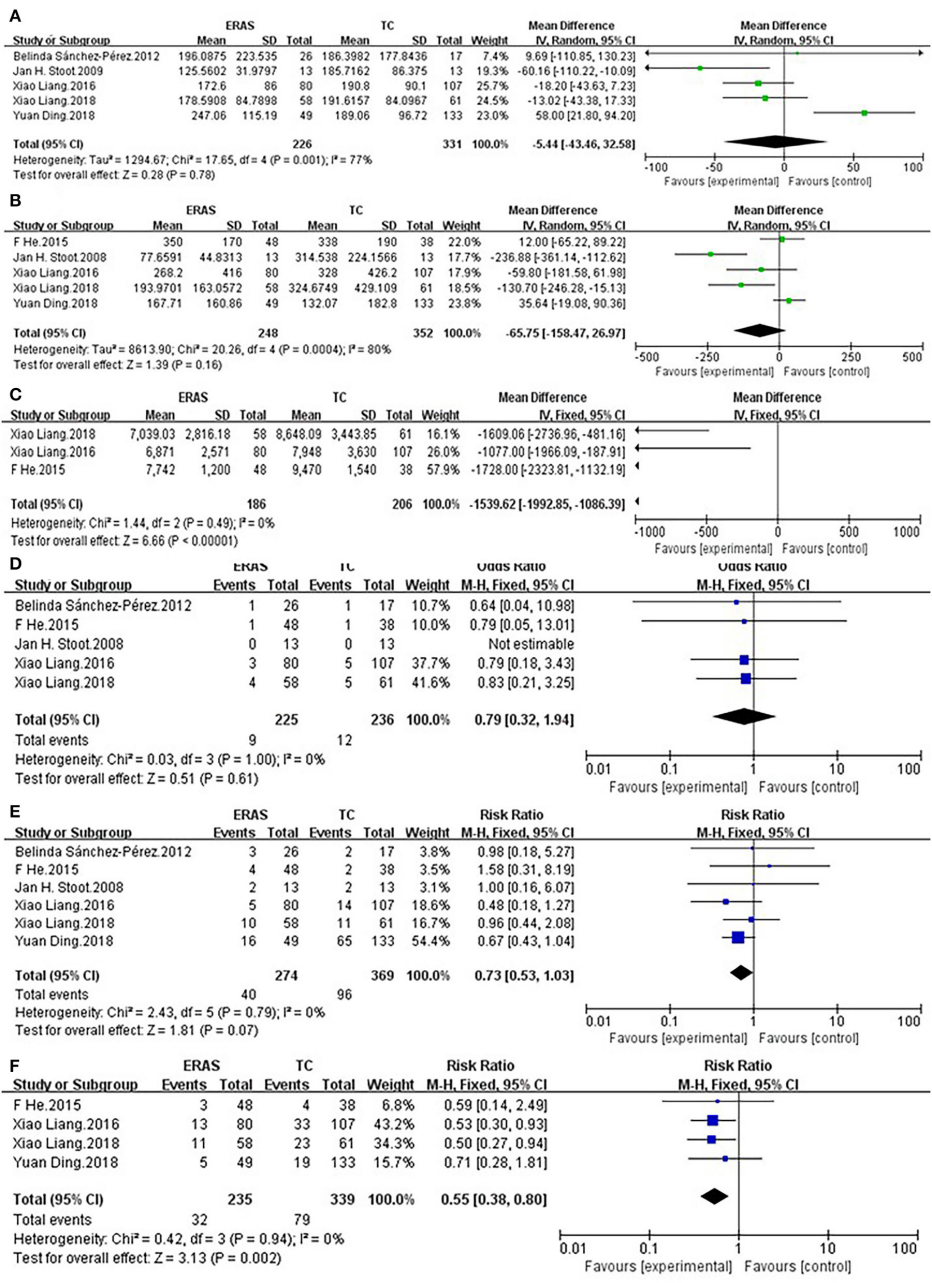
Comparisons of secondary outcomes after ERAS or TC in patients undergoing laparoscopic hepatectomy. The differences in **(A)** operative time, **(B)** intraoperative blood loss, **(C)** cost of hospitalization, **(D)** readmission rate, **(E)** Grade I complication rate, and **(F)** Grade II–V complication rate.

Five studies (25, 27–30) on 557 patients, who underwent ERAS and TC, reported operative time. There was no significant difference in operative time between the ERAS group (*n* = 226) and TC group (*n* = 331), (MD = −5.44, 95% CI = −43.46~32.58, *p* = 0.78). There was significant heterogeneity among the five studies, and a random-effect model was used (*I*^2^ = 77%, *p* = 0.001) (Figure 3A).

#### Intraoperative Blood Loss

No statistical difference existed in intraoperative blood loss between the ERAS group (*n* = 248) and TC group (*n* = 352) (MD= −65.75, 95% CI: = −158.47~26.97, *p* = 0.16). There was significant heterogeneity among the five studies, and a random-effect model was used (*I*^2^ = 80%, *p* = 0.0004) (Figure 3B).

#### Cost of Hospitalization

Although laparoscopic surgery has been widely used, the high cost involved in this surgery when compared with traditional surgery cannot be ignored. Therefore, it was necessary to analyze the difference in hospitalization costs. Three studies (26, 27, 30) reported the cost of hospitalization. The cost of hospitalization of the ERAS group was significantly lower than that of the TC group (MD = −1,539.62, 95% CI = −1992.85~−1086.39, *p* < 0.00001). There was no heterogeneity among the three studies, and a fixed-effect model was used (*I*^2^= 0%, *P* = 0.49) (Figure 3C).

#### Readmission Rate

Five studies (25–27, 29, 30) reported readmission rate. There was no difference in readmission rate between the ERAS group (*n* = 225) and TC group (*n* = 236) (OR = 0.79, 95% CI = 0.32~1.94, *p* = 0.61). No heterogeneity existed among the five studies, and a fixed-effect model was used (*I*^2^ = 0%, *p* = 1.00) (Figure 3D).

#### Grade I Complication Rate

All studies (25–30) reported Grade I complication rate. There was no significant difference in Grade I complication rate between the ERAS group (*n* = 274) and TC group (*n* = 369) (RR = 0.73, 95% CI = 0.53~1.03, *p* = 0.07). There was no heterogeneity among the six studies, and a fixed-effect model was used (*I*^2^ = 0%, *p* = 0.79) (Figure 3E).

#### Grade II–V Complication Rate

Four studies (26–28, 30) reported Grade II–V complication rate. There was significant difference in Grade II–V complication rate between the ERAS group (n = 235) and TC group (*n* = 339) (RR = 0.55, 95% CI = 0.38~0.80, *p* = 0.002). The Grade II–V complication rate of the ERAS group was lower than that of the TC group. There was no heterogeneity among the four studies, and a fixed-effect model was used (*I*^2^ = 0%, *p* = 0.94) (Figure 3F).

### Sensitivity Analysis and Publication Bias

**Figure 4:**
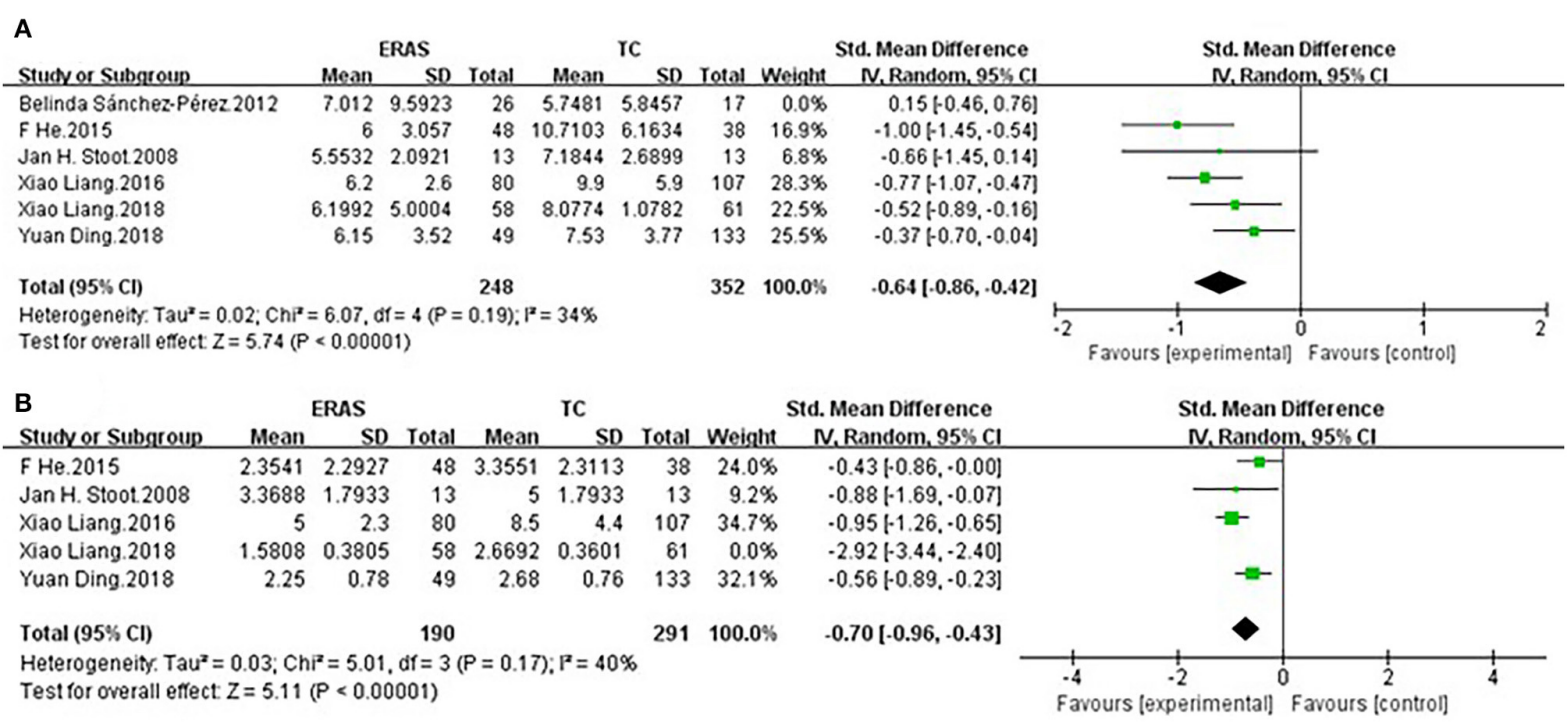
Sensitivity analysis of **(A)** LOS and **(B)** duration to functional recovery.

Sensitivity analysis was performed to evaluate the stability of the duration to functional recovery and LOS. As for LOS, after removing the study by Belinda Sánchez-Pérez et al. (29), high heterogeneity turned into low heterogeneity. We speculated that this alteration was ascribed to the relatively low quality of the study. As for the duration to functional recovery, high heterogeneity turned into low heterogeneity after the removal of the study conducted by Liang et al. (27) (Figure 4).

**Figure 5:**
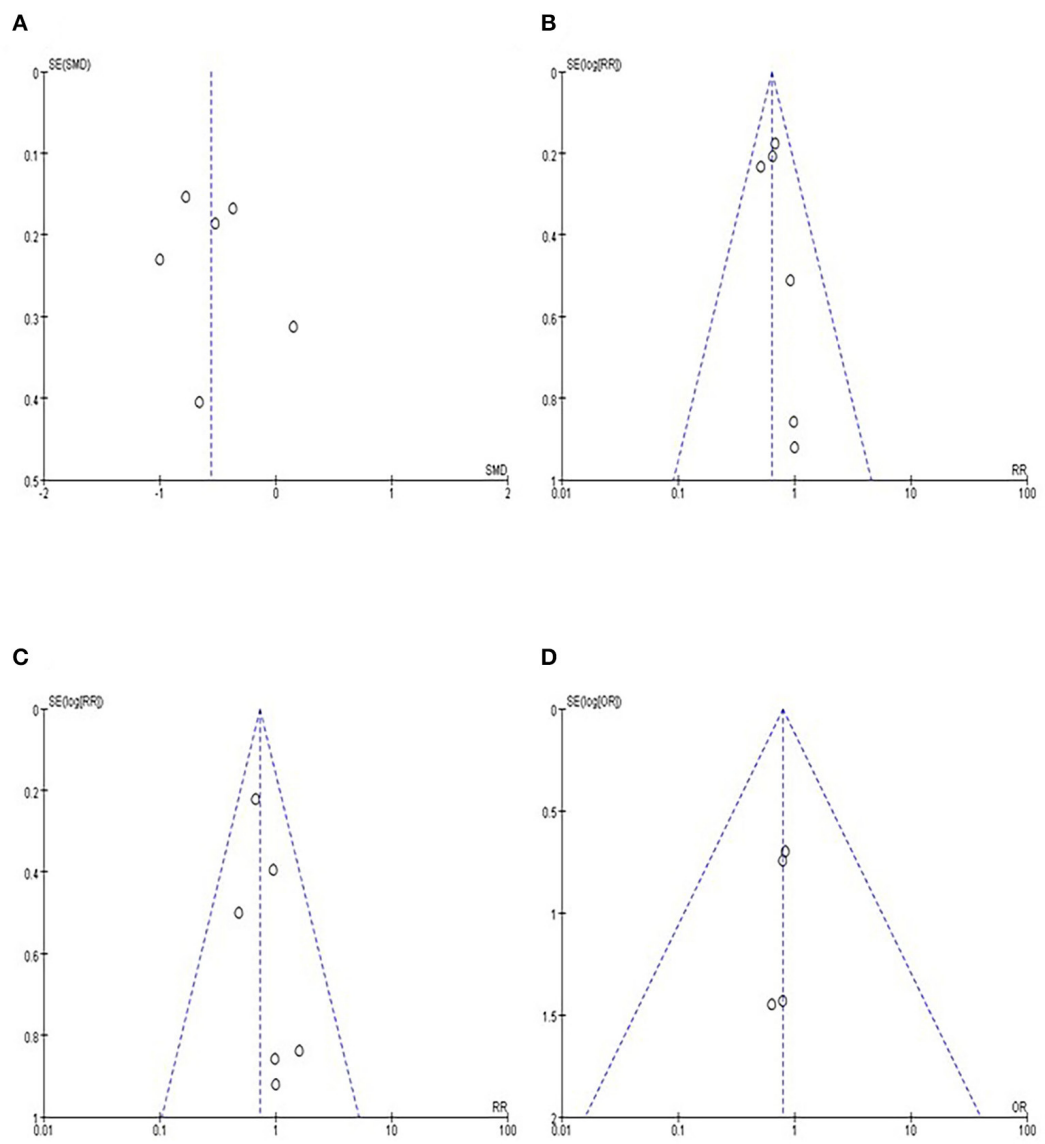
Funnel plot of **(A)** LOS, **(B)** overall postoperative complication rate, **(C)** grade I complication rate, and **(D)** readmission rate.

Funnel plots were used to evaluate the publication bias of the included studies. No significant publication bias was observed (Figure 5).

## Discussion

In the meta-analysis, we observed the preponderance of the ERAS program in laparoscopic hepatectomy in terms of the postoperative safety displayed by lower overall complication rate and Grade II–V complication rate. The ERAS program also manifested better efficacy characterized by lower LOS and duration to functional recovery. Moreover, the ERAS program correlated with a lower cost of hospitalization. In summary, the ERAS program was a promising management during the perioperative period in laparoscopic hepatectomy.

We analyzed the overall postoperative complication rate, Grade I complication rate, and Grade II–V complication rate. The study showed a significant decrease in the postoperative complication rate and Grade II–V complication rate. This indicated that the ERAS program in laparoscopic hepatectomy was safe with less postoperative complications. However, our study showed that there was no significant difference in Grade I complication rate, which wascontrary to the study performed by Yang et al. (31). It was worth affirming that these results were in line with the studies by Ding et al. (28), which reported that the complication in the ERAS group was milder than that in the TC group. The Grade I complication rate might be correlated with the liver surgery itself rather than the ERAS program. Additionally, the complexity of postoperative complications after liver surgery may account for the result.

To assess the efficacy of the ERAS program in laparoscopic hepatectomy, we analyzed the duration of functional recovery and the LOS. The result showed that the time to functional recovery was accelerated in the ERAS group than in the TC group, with high heterogeneity. We performed a sensitivity analysis, which showed that the result was unstable after the removal of the single study conducted by Liang et al. (27). We inferred that this study was the newest among the six studies during which the ERAS program dramatically improved compared with previous studies. Our study also indicated that the LOS was significantly lower in the ERAS group when compared to the TC group, with high heterogeneity. The subsequent sensitivity analysis after the removal of the study conducted by Belinda Sánchez-Pérez et al. (29) showed that the high heterogeneity was caused by the relatively low quality of the excluded study. Even though these two outcomes showed high heterogeneity, our results were in line with others' reports (32, 33). The cost of hospitalization was a crucial factor affecting patients' choices. This meta-analysis showed that the hospitalization cost was significantly decreased than that of TC group. It was obvious that the reduced LOS was accompanied by a lower cost of hospitalization. Our results suggested that the ERAS program can mitigate the financial burden of patients. There were no differences in terms of some parameters (operative time, intraoperative blood loss, and intraoperative blood transfusion rate). These parameters were largely related to the surgical procedure itself and the surgeon's experience and are not the result of differences in the mode of care.

Certain meta-analyses comparing the safety and efficacy between ERAS and TC in liver surgery revealed that ERAS was correlated with lower LOS, complication rate, cost of hospitalization, and shorter duration to functional recovery (22, 33–36). However, those meta-analyses incorporated limited numbers of research. A meta-analysis was performed by Yang et al. (31) containing 580 patients and published in 2016. This meta-analysis with few RCTs addressed the issue that ERAS was superior to non-ERAS in laparoscopic hepatectomy. However, more than two studies with large samples were performed and published after 2016. Besides, high heterogeneity existed in some parameters, including the duration to functional recovery, the cost of hospitalization, and the overall postoperative complication rate. Meanwhile, sensitivity analysis or subgroup analysis was not conducted in the previous study. Compared to the previous results, our results displayed lower heterogeneity in most outcomes, and we conducted a sensitivity analysis in some paramount parameters with high heterogeneity (including duration to functional recovery and LOS). Additionally, our meta-analysis incorporated the latest studies of high quality, contributing to the higher accuracy and convincing of our results. Besides, the overall complication rate and Grade II–V complication rate were significantly lower than that of the TC group. Our results also contained the readmission rate and the pain score, which demonstrated that the ERAS program was safe in laparoscopic hepatectomy.

We searched a current clinical trial comparing the ERAS program with TC in laparoscopic hepatectomy, which was registered in clinicaltrials.gov. An RCT (NCT02533193) was performed to investigate the clinical value of ERAS program in laparoscopic hepatectomy compared with TC by assessing its outcomes and hospital stay days. Though the clinical trial was completed, the results were not available. This clinical trial will provide more robust evidence and outcomes about the ERAS program in laparoscopic hepatectomy. It is important to note that our results will be more precise and credible by adding this latest research.

Admittedly, there were several limitations in the study as follows: (1) Due to the limited number of included studies, more high-quality RCTs should be added to draw accurate conclusions. (2) About half the included studies were non-RCTs, which could increase the risk of publication basis. (3) Although two primary outcomes (duration to functional recovery and LOS) exhibited high heterogeneity, we analyzed the source of heterogeneity, and the result was in line with most meta-analyses.

## Conclusions

The ERAS group had shorter LOS and duration to functional recovery, and less cost of hospitalization. The incidence of overall complication rate and Grade II–V complication rate was significantly lower in the ERAS group. There was no difference in operative time, intraoperative blood loss, intraoperative blood transfusion rate, Grade I complication rate, and readmission rate. Above all, it is reasonable that the ERAS program should be recommended in laparoscopic hepatectomy.

## Data Availability Statement

The original contributions presented in the study are included in the article/Supplementary Material, further inquiries can be directed to the corresponding author.

## Author Contributions

XueyinZ, XueyiZ, JC, and MC wrote this article. JH, WT, SL, SJ, ZL, BZ, XF, and JS reviewed this article. All authors read and approved the final manuscript.

## Funding

This work was supported by the Scientific Research Fund of Zhejiang Provincial Education Department (Y202148325).

## Conflict of Interest

The authors declare that the research was conducted in the absence of any commercial or financial relationships that could be construed as a potential conflict of interest.

## Publisher's Note

All claims expressed in this article are solely those of the authors and do not necessarily represent those of their affiliated organizations, or those of the publisher, the editors and the reviewers. Any product that may be evaluated in this article, or claim that may be made by its manufacturer, is not guaranteed or endorsed by the publisher.

## Abbreviations

CIs: confidence intervals
ERAS: enhanced recovery after surgery
LOS: length of hospital stay
OR: odds ratio
RCTs: randomized clinical trials
RR: risk ratio
SMD: standard mean difference
TC: traditional care
WMD: weighted mean difference.
