# Supplementary material for: Enhanced Recovery Care vs. Traditional Care in Laparoscopic Hepatectomy: A Systematic Review and Meta-Analysis
Source: Front Surg. 2022 Mar 22;9:850844. doi: 10.3389/fsurg.2022.850844 (PMC8980421; doi:10.3389/fsurg.2022.850844)
Supplement: Supplementary file 1 [file Table_1.DOCX]

The Risk Of Bias In Non-randomized Studies – of Interventions (ROBINS-I) assessment tool

(version for cohort-type studies)

**Version 19 September 2016**


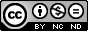


This work is licensed under a [Creative Commons Attribution-NonCommercial-NoDerivatives 4.0 International License](http://creativecommons.org/licenses/by-nc-nd/4.0/).

# ROBINS-I tool (Stage I): At protocol stage

## Specify the review question

| Participants | Patients who received Laparoscopic Hepatectomy |
| --- | --- |
| Experimental intervention | ERAS |
| Comparator | TC |
| Outcomes | All outcomes included in our meta-analysis |

## List the confounding domains relevant to all or most studies

| D1-D7 |
| --- |

## List co-interventions that could be different between intervention groups and that could impact on outcomes

| The ERAS protocol is not uniform in all studies. |
| --- |

# ROBINS-I tool (Stage II): For each study

## Specify a target randomized trial specific to the study

| Design | Individually randomized |
| --- | --- |
| Participants | Patients who received Laparoscopic Hepatectomy |
| Experimental intervention | ERAS |
| Comparator | TC |

## Is your aim for this study…?

| √ | to assess the effect of *assignment to* intervention |
| --- | --- |
| □ | to assess the effect of *starting and adhering to* intervention |

## Specify the outcome

Specify which outcome is being assessed for risk of bias (typically from among those earmarked for the Summary of Findings table). Specify whether this is a proposed benefit or harm of intervention.

| LOS: harm; duration to function recovery: benefit; harm; Overall postoperative complication rate: benefit; Operative time; Intraoperative blood loss: benefit ; Cost of hospitalization: benefit ;Readmission rate: harm ; Grade I complication rate: benefit ; Grade II-V complication rate: benefit. |
| --- |

## Specify the numerical result being assessed

In case of multiple alternative analyses being presented, specify the numeric result (e.g., RR = 1.52 (95% CI 0.83 to 2.77) and/or a reference (e.g. to a table, figure or paragraph) that uniquely defines the result being assessed.

| hospital stay (SMD=-0.56, 95%CI=-0.83~-0.28, *P*<0.0001); duration to function recovery (SMD=-1,14, 95%CI=-1.92~-0.37, *P*=0.004); cost of hospitalization (MD=-1539.62, 95%CI=-1992.85~-1086.39, *P*<0.00001); overall postoperative complication rate (RR=0.64, 95%CI=0.51~0.80, *P*<0.0001); Grade II-V complication rate (RR=0.55, 95%CI=0.38~0.80, *P*=0.002); intraoperative blood loss (MD=-65.75, 95%CI=-158.47~26.97, *P*=0.16); perative time (MD=-5.44, 95%CI=-43.46~32.58, *P*=0.78), intraoperative blood transfusion rate (OR=0.71, 95%CI=0.41~1.22, *P*=0.22); Grade I complication rate (RR=0.73, 95%CI=0.53~1.03, *P*=0.07). |
| --- |

## Preliminary consideration of confounders

Complete a row for each important confounding domain (i) listed in the review protocol; and (ii) relevant to the setting of this particular study, or which the study authors identified as potentially important.

#### “Important” confounding domains are those for which, in the context of this study, adjustment is expected to lead to a clinically important change in the estimated effect of the intervention. “Validity” refers to whether the confounding variable or variables fully measure the domain, while “reliability” refers to the precision of the measurement (more measurement error means less reliability).

| **(i) Confounding domains listed in the review protocol** | | | | |
| --- | --- | --- | --- | --- |
| Confounding domain | Measured variable(s) | Is there evidence that controlling for this variable was unnecessary? * | Is the confounding domain measured validly and reliably by this variable (or these variables)? | OPTIONAL: Is failure to adjust for this variable (alone) expected to favour the experimental intervention or the comparator? |
| No information | No information | No information | No information | No information |
|  |  |  |  |  |
|  |  |  |  |  |
|  |  |  |  |  |
|  |  |  |  |  |
|  |  |  |  |  |
|  |  |  |  |  |
|  |  |  |  |  |
|  |  |  |  |  |

| **(ii) Additional confounding domains relevant to the setting of this particular study, or which the study authors identified as important** | | | | |
| --- | --- | --- | --- | --- |
| Confounding domain | Measured variable(s) | Is there evidence that controlling for this variable was unnecessary?* | Is the confounding domain measured validly and reliably by this variable (or these variables)? | OPTIONAL: Is failure to adjust for this variable (alone) expected to favour the experimental intervention or the comparator? |
| No information | No information | No information | No information | No information |
|  |  |  |  |  |
|  |  |  |  |  |
|  |  |  |  |  |
|  |  |  |  |  |
|  |  |  |  |  |
|  |  |  |  |  |
|  |  |  |  |  |
|  |  |  |  |  |
|  |  |  |  |  |

* In the context of a particular study, variables can be demonstrated not to be confounders and so not included in the analysis: (a) if they are not predictive of the outcome; (b) if they are not predictive of intervention; or (c) because adjustment makes no or minimal difference to the estimated effect of the primary parameter. Note that “no statistically significant association” is not the same as “not predictive”.

## Preliminary consideration of co-interventions

Complete a row for each important co-intervention (i) listed in the review protocol; and (ii) relevant to the setting of this particular study, or which the study authors identified as important.

#### “Important” co-interventions are those for which, in the context of this study, adjustment is expected to lead to a clinically important change in the estimated effect of the intervention.

| **(i) Co-interventions listed in the review protocol** | | |
| --- | --- | --- |
| Co-intervention | Is there evidence that controlling for this co-intervention was unnecessary (e.g. because it was not administered)? | Is presence of this co-intervention likely to favour outcomes in the experimental intervention or the comparator |
| No information | No information | No information |
|  |  | Favour experimental / Favour comparator / No information |
|  |  | Favour experimental / Favour comparator / No information |
|  |  | Favour experimental / Favour comparator / No information |

| **(ii) Additional co-interventions relevant to the setting of this particular study, or which the study authors identified as important** | | |
| --- | --- | --- |
| Co-intervention | Is there evidence that controlling for this co-intervention was unnecessary (e.g. because it was not administered)? | Is presence of this co-intervention likely to favour outcomes in the experimental intervention or the comparator |
| No information | No information | No information |
|  |  | Favour experimental / Favour comparator / No information |
|  |  | Favour experimental / Favour comparator / No information |
|  |  | Favour experimental / Favour comparator / No information |

## Risk of bias assessment

Responses underlined in green are potential markers for low risk of bias, and responses in red are potential markers for a risk of bias. Where questions relate only to sign posts to other questions, no formatting is used.

|  | **Signalling questions** | **Description** | **Response options** |
| --- | --- | --- | --- |
| **Bias due to confounding** | | | |
|  | 1.1 Is there potential for confounding of the effect of intervention in this study?  **If N/PN to 1.1:** the study can be considered to be at low risk of bias due to confounding and no further signalling questions need be considered | Answer: PN | Y / PY / PN / N |
|  | **If Y/PY to 1.1**: determine whether there is a need to assess time-varying confounding: |  |  |
|  | 1.2. Was the analysis based on splitting participants’ follow up time according to intervention received?  **If N/PN**, answer questions relating to baseline confounding (1.4 to 1.6)  **If Y/PY**, go to question 1.3. | Answer: N | NA / Y / PY / PN / N / NI |
|  | 1.3. Were intervention discontinuations or switches likely to be related to factors that are prognostic for the outcome?  **If N/PN**, answer questions relating to baseline confounding (1.4 to 1.6)  **If Y/PY**, answer questions relating to both baseline and time-varying confounding (1.7 and 1.8) |  | NA / Y / PY / PN / N / NI |

|  | **Questions relating to baseline confounding only** | | |
| --- | --- | --- | --- |
|  | 1.4. Did the authors use an appropriate analysis method that controlled for all the important confounding domains? | Answer: Y Statistical analysis was made with SPSS version 15.0 for  Windows (SPSS, Chicago, IL, United States). Continuous variables (age, sex, post-operative stay and duration of surgery) were defined by median (rank) or mean and analyzed with the Mann-Whitney U test. | NA / Y / PY / PN / N / NI |
|  | 1.5. **If Y/PY to 1.4**: Were confounding domains that were controlled for measured validly and reliably by the variables available in this study? | Answer: PY | NA / Y / PY / PN / N / NI |
|  | 1.6. Did the authors control for any post-intervention variables that could have been affected by the intervention? | Answer: PY | NA / Y / PY / PN / N / NI |
|  | **Questions relating to baseline and time-varying confounding** | |  |
|  | 1.7. Did the authors use an appropriate analysis method that controlled for all the important confounding domains and for time-varying confounding? | Answer: Y Qualitative variables (use of drains, conversion, transfusion, intensive cares, readmissions and morbidity) were defined by percentages and analyzed by χ2 test with Yates correction and Fisher’s exact test. | NA / Y / PY / PN / N / NI |
|  | 1.8. **If Y/PY to 1.7**: Were confounding domains that were controlled for measured validly and reliably by the variables available in this study? | Answer: PY | NA / Y / PY / PN / N / NI |
|  | **Risk of bias judgement** | Moderate | Low / Moderate / Serious / Critical / NI |
|  | Optional: What is the predicted direction of bias due to confounding? | Favours experimental | Favours experimental / Favours comparator / Unpredictable |

| **Bias in selection of participants into the study** | | | |
| --- | --- | --- | --- |
|  | 2.1. Was selection of participants into the study (or into the analysis) based on participant characteristics observed after the start of intervention?  **If N/PN to 2.1:** go to 2.4 | Answer: N | Y / PY / PN / N / NI |
|  | 2.2. **If Y/PY to 2.1**: Were the post-intervention variables that influenced selection likely to be associated with intervention?  2.3 **If Y/PY to 2.2**: Were the post-intervention variables that influenced selection likely to be influenced by the outcome or a cause of the outcome? |  | NA / Y / PY / PN / N / NI  NA / Y / PY / PN / N / NI |
|  | 2.4. Do start of follow-up and start of intervention coincide for most participants? | Answer: PY | Y / PY / PN / N / NI |
|  | 2.5. **If Y/PY to 2.2 and 2.3, or N/PN to 2.4**: Were adjustment techniques used that are likely to correct for the presence of selection biases? |  | NA / Y / PY / PN / N / NI |
|  | **Risk of bias judgement** | Low | Low / Moderate / Serious / Critical / NI |
|  | Optional: What is the predicted direction of bias due to selection of participants into the study? | Favours experimental | Favours experimental / Favours comparator / Towards null /Away from null / Unpredictable |

| **Bias in classification of interventions** | | | |
| --- | --- | --- | --- |
|  | 3.1 Were intervention groups clearly defined? | Answer: Y Basically, our FT program for laparoscopic liver surgery consisted in: (1) reduction of indications of drains after surgical procedure; (2) changing location of patients in the early post-operative period, considering not necessary ICU admission for patients without severe morbidity, undergoing atypical resections or typical up to two segments, without transfusions and without/less than 30 min vascular clamping; (3) obviate necessity of a nasogastric tube; (4) prescription of early oral intake and ambulation (introduced the same evening of surgery, 6-8 h after); and (5) optimization of analgesia, according with Anaesthesia Department suggestions, with metamizol/8 h and paracetamol/8 h alternating, initially intravenous and oral afterwards, avoiding opioids. A description of the whole FT program is shown in Table 1. | Y / PY / PN / N / NI |
|  | 3.2 Was the information used to define intervention groups recorded at the start of the intervention? | Answer: N | Y / PY / PN / N / NI |
|  | 3.3 Could classification of intervention status have been affected by knowledge of the outcome or risk of the outcome? | Answer: N | Y / PY / PN / N / NI |
|  | **Risk of bias judgement** | Answer: Moderate | Low / Moderate / Serious / Critical / NI |
|  | Optional: What is the predicted direction of bias due to classification of interventions? | Favours experimental | Favours experimental / Favours comparator / Towards null /Away from null / Unpredictable |

| **Bias due to deviations from intended interventions** | | | |
| --- | --- | --- | --- |
|  | **If your aim for this study is to assess the effect of assignment to intervention, answer questions 4.1 and 4.2** | |  |
|  | 4.1. Were there deviations from the intended intervention beyond what would be expected in usual practice? | Answer: N | Y / PY / PN / N / NI |
|  | 4.2. **If Y/PY to 4.1**: Were these deviations from intended intervention unbalanced between groups *and* likely to have affected the outcome? |  | NA / Y / PY / PN / N / NI |
|  | **If your aim for this study is to assess the effect of starting and adhering to intervention, answer questions 4.3 to 4.6** | |  |
|  | 4.3. Were important co-interventions balanced across intervention groups? | Answer: PN | Y / PY / PN / N / NI |
|  | 4.4. Was the intervention implemented successfully for most participants? | Answer: PY | Y / PY / PN / N / NI |
|  | 4.5. Did study participants adhere to the assigned intervention regimen? | Answer: Y Follow-up was accomplished by the same surgeon the following day after leaving the hospital (by telephone) and 7 d after discharge. | Y / PY / PN / N / NI |
|  | 4.6. **If N/PN to 4.3, 4.4 or 4.5**: Was an appropriate analysis used to estimate the effect of starting and adhering to the intervention? | Answer: N | NA / Y / PY / PN / N / NI |
|  | **Risk of bias judgement** | Serious | Low / Moderate / Serious / Critical / NI |
|  | Optional: What is the predicted direction of bias due to deviations from the intended interventions? | Favours experimental | Favours experimental / Favours comparator / Towards null /Away from null / Unpredictable |

| **Bias due to missing data** | | | |
| --- | --- | --- | --- |
|  | 5.1 Were outcome data available for all, or nearly all, participants? | Answer: PN | Y / PY / PN / N / NI |
|  | 5.2 Were participants excluded due to missing data on intervention status? | Answer: PN | Y / PY / PN / N / NI |
|  | 5.3 Were participants excluded due to missing data on other variables needed for the analysis? | Answer: PN | Y / PY / PN / N / NI |
|  | 5.4 **If PN/N to 5.1, or Y/PY to 5.2 or 5.3**: Are the proportion of participants and reasons for missing data similar across interventions? | Answer: PY | NA / Y / PY / PN / N / NI |
|  | 5.5 **If PN/N to 5.1, or Y/PY to 5.2 or 5.3**: Is there evidence that results were robust to the presence of missing data? | Answer: PN | NA / Y / PY / PN / N / NI |
|  | **Risk of bias judgement** | Serious | Low / Moderate / Serious / Critical / NI |
|  | Optional: What is the predicted direction of bias due to missing data? | Favours experimental | Favours experimental / Favours comparator / Towards null /Away from null / Unpredictable |

| **Bias in measurement of outcomes** | | | |
| --- | --- | --- | --- |
|  | 6.1 Could the outcome measure have been influenced by knowledge of the intervention received? | Answer: N | Y / PY / PN / N / NI |
|  | 6.2 Were outcome assessors aware of the intervention received by study participants? | Answer: PY | Y / PY / PN / N / NI |
|  | 6.3 Were the methods of outcome assessment comparable across intervention groups? | Answer: PY | Y / PY / PN / N / NI |
|  | 6.4 Were any systematic errors in measurement of the outcome related to intervention received? | Answer: PN | Y / PY / PN / N / NI |
|  | **Risk of bias judgement** | Moderate | Low / Moderate / Serious / Critical / NI |
|  | Optional: What is the predicted direction of bias due to measurement of outcomes? | Favours experimental | Favours experimental / Favours comparator / Towards null /Away from null / Unpredictable |

| **Bias in selection of the reported result** | | | |
| --- | --- | --- | --- |
|  | Is the reported effect estimate likely to be selected, on the basis of the results, from... |  |  |
|  | 7.1. ... multiple outcome *measurements* within the outcome domain? | Favours N | Y / PY / PN / N / NI |
|  | 7.2 ... multiple *analyses* of the intervention-outcome relationship? | Favours N | Y / PY / PN / N / NI |
|  | 7.3 ... different *subgroups*? | Favours PN | Y / PY / PN / N / NI |
|  | **Risk of bias judgement** | Low | Low / Moderate / Serious / Critical / NI |
|  | Optional: What is the predicted direction of bias due to selection of the reported result? | Favours comparator | Favours experimental / Favours comparator / Towards null /Away from null / Unpredictable |

| **Overall bias** | | | |
| --- | --- | --- | --- |
|  | **Risk of bias judgement** | Serious | Low / Moderate / Serious / Critical / NI |
|  | Optional: What is the overall predicted direction of bias for this outcome? | Favours experimental | Favours experimental / Favours comparator / Towards null /Away from null / Unpredictable |


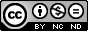


This work is licensed under a [Creative Commons Attribution-NonCommercial-NoDerivatives 4.0 International License](http://creativecommons.org/licenses/by-nc-nd/4.0/).
