# Supplementary material for: Enhanced Recovery Care vs. Traditional Care in Laparoscopic Hepatectomy: A Systematic Review and Meta-Analysis
Source: Front Surg. 2022 Mar 22;9:850844. doi: 10.3389/fsurg.2022.850844 (PMC8980421; doi:10.3389/fsurg.2022.850844)
Supplement: Supplementary file 2 [file Data_Sheet_1.PDF]

frontiers

(A)

Comparison of hospital stay for [health problem]

Patient or population: patients with [health problem]
Settings:
Intervention: Comparison of hospital stay

Outcomes	Illustrative comparative risks* (95% CI)		Relative effect (95% CI)	No of Participants (studies)	Quality of the evidence (GRADE)	Comments
	Assumed risk	Corresponding risk				
	Control	Comparison of hospital stay				
**Comparison of hospital stay**		The mean comparison of hospital stay in the intervention groups was **0.56 standard deviations lower** (0.83 to 0.28 lower)		643 (6 studies)	⊕⊕⊕⊕ **high**	SMD -0.56 (-0.83 to -0.28)

*The basis for the **assumed risk** (e.g. the median control group risk across studies) is provided in footnotes. The **corresponding risk** (and its 95% confidence interval) is based on the assumed risk in the comparison group and the **relative effect** of the intervention (and its 95% CI).

CI: Confidence interval;

GRADE Working Group grades of evidence
High quality: Further research is very unlikely to change our confidence in the estimate of effect.
Moderate quality: Further research is likely to have an important impact on our confidence in the estimate of effect and may change the estimate.
Low quality: Further research is very likely to have an important impact on our confidence in the estimate of effect and is likely to change the estimate.
Very low quality: We are very uncertain about the estimate.

(B)

Comparation of Duration to function recovery for [health problem]

Patient or population: patients with [health problem]
Settings:
Intervention: Duration to function recovery

Outcomes	Illustrative comparative risks* (95% CI)		Relative effect (95% CI)	No of Participants (studies)	Quality of the evidence (GRADE)	Comments
	Assumed risk	Corresponding risk				
	Control	Duration to function recovery				
**Time to first flatus**		The mean time to first flatus in the intervention groups was **1.14 standard deviations lower** (1.92 to 0.37 lower)		600 (5 studies)	⊕⊕⊕⊕ **high**	SMD -1.14 (-1.92 to -0.37)

*The basis for the **assumed risk** (e.g. the median control group risk across studies) is provided in footnotes. The **corresponding risk** (and its 95% confidence interval) is based on the assumed risk in the comparison group and the **relative effect** of the intervention (and its 95% CI).

CI: Confidence interval;

GRADE Working Group grades of evidence
High quality: Further research is very unlikely to change our confidence in the estimate of effect.
Moderate quality: Further research is likely to have an important impact on our confidence in the estimate of effect and may change the estimate.
Low quality: Further research is very likely to have an important impact on our confidence in the estimate of effect and is likely to change the estimate.
Very low quality: We are very uncertain about the estimate.

(C)

Comparison of postoperative complication rate for [health problem]

Patient or population: patients with [health problem]
Settings:
Intervention: Comparison of postoperative complication rate

Outcomes	Illustrative comparative risks* (95% CI)		Relative effect (95% CI)	No of Participants (studies)	Quality of the evidence (GRADE)	Comments
	Assumed risk	Corresponding risk				
	Control	Comparison of postoperative complication rate				
Comparison of postoperative complication rate	Study population		RR 0.64 (0.51 to 0.8)	643 (6 studies)	⊕⊕⊕⊕ high	
	474 per 1000	304 per 1000 (242 to 379)				
	Moderate					
	299 per 1000	191 per 1000 (152 to 239)				

*The basis for the **assumed risk** (e.g. the median control group risk across studies) is provided in footnotes. The **corresponding risk** (and its 95% confidence interval) is based on the assumed risk in the comparison group and the **relative effect** of the intervention (and its 95% CI).

CI: Confidence interval; **RR:** Risk ratio;

GRADE Working Group grades of evidence
High quality: Further research is very unlikely to change our confidence in the estimate of effect.
Moderate quality: Further research is likely to have an important impact on our confidence in the estimate of effect and may change the estimate.
Low quality: Further research is very likely to have an important impact on our confidence in the estimate of effect and is likely to change the estimate.
Very low quality: We are very uncertain about the estimate.

Supplementary Figure 2. Quantitative rating of (A) LOS, (B) Duration to function recovery, (C) postoperative complication rate.
