# Supplementary figures and images for: Enhanced Recovery Care vs. Traditional Care in Laparoscopic Hepatectomy: A Systematic Review and Meta-Analysis
Source: Front Surg. 2022 Mar 22;9:850844. doi: 10.3389/fsurg.2022.850844 (PMC8980421; doi:10.3389/fsurg.2022.850844)

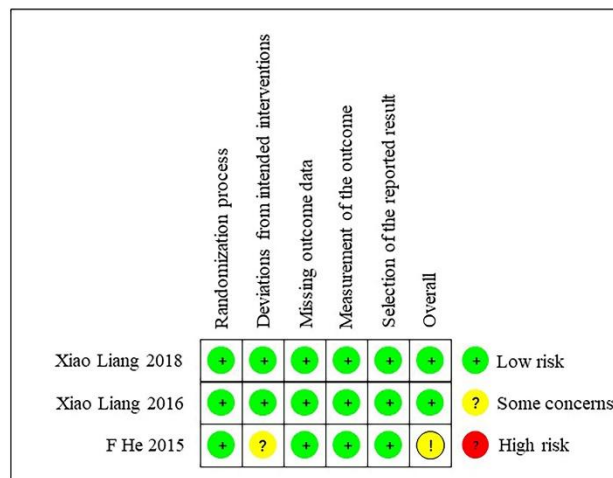

**Supplementary Figure 1.** Risk of bias graph of all RCTs included in the meta-analysis.

Supplement: Supplementary file 3 [file Data_Sheet_2.PDF]
